# Cognitive Stimulation as Alternative Treatment to Improve Psychological Disorders in Patients with Mild Cognitive Impairment

**DOI:** 10.3390/jcm11143947

**Published:** 2022-07-07

**Authors:** María del Carmen Carcelén-Fraile, Ana María Llera-DelaTorre, Agustín Aibar-Almazán, Diego Fernando Afanador-Restrepo, Mateo Baena-Marín, Fidel Hita-Contreras, Vânia Brandão-Loureiro, Patricia Alexandra García-Garro, Yolanda Castellote-Caballero

**Affiliations:** 1 Department of Health Sciences, Faculty of Health Sciences, University of Jaén, 23071 Jaén, Spain; mccf0004@red.ujaen.es (M.d.C.C.-F.); llera30@hotmail.com (A.M.L.-D.); fhita@ujaen.es (F.H.-C.); mycastel@ujaen.es (Y.C.-C.); 2 Faculty of Health Sciences, University Foundation of the Área Andina, Pereira 660004, Colombia; dafanador4@areandina.edu.co (D.F.A.-R.); mbaena@areandina.edu.co (M.B.-M.); 3 Escola Superior de Educação, Instituto Politécnico de Beja, 7800-295 Beja, Portugal; vloureiro@ipbeja.pt; 4 GIP Pedagogy Research Group, Faculty of Distance and Virtual Education, Antonio José Camacho University Institution, Santiago de Cali 760016, Colombia; palexandragarcia@admon.uniajc.edu.co

**Keywords:** cognitive training, mild cognitive impairment, quality of life, anxiety, depression

## Abstract

(1) Background: Mild cognitive impairment is becoming one of the most common clinical manifestations affecting older people. For this reason, developing non-pharmacological strategies to help improve or maintain the physical condition of patients with mild dementia has become a priority. Therefore, the objective of this study is to provide evidence about the effects of a cognitive stimulation program on cognitive performance, anxiety, depression, and quality of life in people with mild cognitive impairment (MCI) and aged > 70. (2) Methods: This study is a randomized clinical trial. A total of 72 elderly people with MCI participated: 35 in the control group who did not receive any intervention and 36 in the experimental group who received a cognitive stimulation program for 12 weeks. Cognitive performance, depression, anxiety and health-related quality of life (HRQoL) were measured using the Mini-Mental State Examination (MMSE), the Yesavage Geriatric Depression Scale, the Hamilton Rating Scale, and the SF-12, respectively. (3) Results: In the experimental group, significant results were obtained on cognitive performance, depression, anxiety and general health, emotional role, social functioning, vitality, mental health and mental component summary domains of the SF-12. (4) Conclusions: a cognitive stimulation program of 12 weeks improves cognitive performance, anxiety, depression, and HRQoL in people with MCI aged > 70.

## 1. Introduction

Currently, not only worldwide, but also within Europe, and more specifically in Spain, the average life expectancy of the population is increasing, leading to important changes such as the aging of the population [1]. According to data from the National Institute of Statistics, it can be expected that by the year 2050, the population aged over 65 will represent 36.8% of the total population, placing Spain as one of the European countries with the largest elderly population [2]. An important part of the aging process is the decline in cognitive function that can lead to mild cognitive impairment (MCI). This aging, together with possible common comorbidities such as poor glycemic control, episodes of hypoglycemia and hyperglycemia or vascular complications can cause a progression of mild cognitive impairment and its possible conversion to dementia [3]. In addition, it has been shown that the glycemic variable and hypertension are associated with dementia-like diseases such as progressive supranuclear palsy, Alzheimer’s disease, and corticobasal syndrome [4,5]. MCI is known as a neurological disorder that is somewhere between normal functioning and dementia [6] and is defined by the WHO as a disorder characterized by memory impairment, learning difficulties, and reduced ability to concentrate on a task for more than brief periods [7]. The prevalence of MCI increases with age and has an incidence ranging from 21.5 to 71.3 cases per 1000 inhabitants/year [8]. This is associated with an increased risk of dementia, as well as adverse health outcomes, such as functional limitations and disability [9].

People with MCI suffer a reduction in their cognitive functioning, resulting in alterations in mood or behavior [10]. One of the most challenging disorders is depression, which could be a risk factor for progression from MCI to dementia [11]. In addition, it has been shown that people with MCI are more likely to suffer from depressive disorders than people without MCI [12]. Moreover, it has been suggested that anxiety is also associated with an increased risk of dementia, with estimated prevalence rates ranging from 8% to 71% in this population [13]. This is because the individual’s concern about the loss of cognitive function contributes to a heightened state of anxiety [14].

This MCI in the elderly also affects other variables such as quality of life (HRQoL), which tends to worsen as the impairment increases, affecting their physical and mental health, social relationships, and participation in activities [15]. Quality of life is a key concept in understanding the subjective dimension of MCI and the impact it has on patients [16]. The scientific evidence comparing the quality of life of people with and without cognitive impairment is inconsistent; some report that there is no difference between them [17,18], while others report a worse quality of life for people diagnosed with MCI in nearly all areas [19,20]. However, most studies focus on the concept of MCI as a risk factor for dementia rather than on the direct effects of MCI on the elderly [21,22]. In addition, these also refer to very selective samples from memory clinics or nursing homes, leaving no evidence in the community resident population [17].

Therefore, it is necessary to address this public health problem, and intervention strategies that can delay or prevent the risk of dementia are needed [23]. Within these strategies are non-pharmacological interventions that can be fundamental to prevent or delay cognitive impairment and with it, functional disability [24]. Cognitive stimulation is a cognitive training modality that consists of the participation of cognitive activities in a group, proposed to improve cognitive and social activity. This modality is composed of activities such as memorization, orientation and leisure activities and is based on unimodal interventions, i.e., they are focused on one domain [25].

Considering the previous points, the aim of this study is to evaluate the effectiveness of a cognitive stimulation program on cognitive performance, anxiety, depression and HRQoL in people with MCI aged > 70.

## 2. Materials and Methods

### 2.1. Study Design

A randomized controlled clinical trial that analyzed the benefits of a cognitive stimulation program on cognitive performance, anxiety, depression and HRQoL in people aged > 70 with MCI (NCT04663256) was performed. All persons who participated in this work signed informed consent in accordance with the Declaration of Helsinki, good practices and applicable laws and regulations. This study was also approved by the Ethics Committee of the University of Jaén (20/1.SEPT.TFM).

### 2.2. Participants

**Figure 1:**
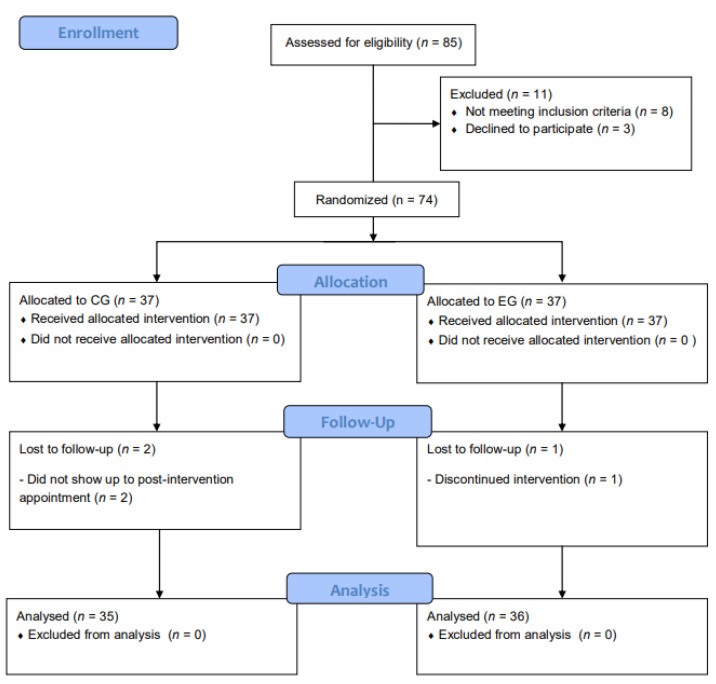
Flow diagram of study design.

All participants were recruited from two day care centers in the city of Jaén (Spain). Out of a total of 85 participants who were initially contacted, 74 met the eligibility criteria and agreed to participate in the study (Figure 1). Individuals who were included in the study: (i) had self-reported memory complaints; scored between ≥24 to ≤26 on the Mini-Mental State Examination (MMSE) and scored between ≥18 to ≤26 points on the Montreal Cognitive Assessment (MoCA); (ii) were 70 years of age or older; (iii) without mobility dependence, and (iv) were able to understand and complete each of the self-administered questionnaires. Exclusion criteria were, (i) had a diagnosis of dementia; (ii) were visually impaired; (iii) had some comorbidity such as cardiovascular disease or diabetes; (iv) diagnosed with a neurodegenerative disease, (e.g., Parkinson’s disease), and (v) were enrolled in a cognitive program.

### 2.3. Randomization

Participants who met the eligibility criteria were randomly assigned to an experimental group (EG) consisting of a total of 37 people and a control group (CG) with another 37 participants. This randomization was carried out using a table of random numbers produced by a computer. Subsequently, sealed opaque envelopes were used and group assignment was performed by an independent researcher unrelated to participant selection, intervention or data collection.

### 2.4. Intervention

The intervention was carried out from January to April 2021. Participants included in the experimental group followed a cognitive stimulation program consisting of 3 sessions per week for 12 weeks, for a total of 36 sessions. Each session was aimed at training a cognitive domain, had a duration of 60 min and consisted of three parts: (i) orientation to reality: temporal, spatial and personal orientation exercises; (ii) individual exercises of a cognitive domain: (a) memory: association, categorization and memorization exercises; (b) language: verbal comprehension and expression, literacy and verbal fluency exercises; (c) calculation: arithmetic operations and mathematical problem solving; (d) praxis and gnosis: fine motor skills and auditory, tactile, spatial and visual recognition and attention; (e) executive functions: planning and organization capacity exercises; (iii) group correction of practical exercises. All patients were trained in the same tasks in each session. On the other hand, the participants assigned to the control group maintained their daily activities and received the usual care according to the clinical orientation, which included counseling and a series of recommendations aimed at promoting their mental health.

### 2.5. Outcomes

All variables were measured before and after the intervention. Descriptive characteristics such as age, sex, marital status (single, married, widowed/separated) and educational level (no studies, primary, secondary, university studies) were collected through questionnaires administered by qualified personnel. Weight and height were assessed with a precision digital scale from 100 g to 130 kg (Tefal, Sarcelles, France) and an adult height scale (Asimed T201-T4, Guayaquil, Ecuador).

#### 2.5.1. Cognitive Performance

Mini-Mental State Examination is used to measure global cognitive function and is designed to detect severe cognitive deficits [26]. This test consists of a total of 30 items that comprise 5 cognitive areas: orientation, registration, language, memory and calculation. The total score for this test is 30 points, with higher scores indicating better overall cognition on the part of the subject. Reference scores are: dementia: 9 to 12; deterioration: from 12 to 24; pathological suspicion: 24 or more; normal: 27 or more.

#### 2.5.2. Cognitive Impairment

The Montreal Cognitive Assessment is a short test composed of 12 items that assesses cognitive function through 7 cognitive domains: visuospatial and executive function (task B of tracing (1 point), copy of the cube (1 point) and clock drawing (3 points)), denomination (3 points), attention (forward/towards behind), digit interval (2 points), vigilance/tapping (1 point) and subtraction from series (3 points), language (repetition of sentences (2 points) and verbal fluency (1 point)), abstraction (the element 2-element verbal abstraction, total of 2 points), delayed recall/short-term memory (5 points) and orientation (6 points). The maximum MoCA total score is 30, and values ≥ 26 indicate normal cognitive functioning [27].

#### 2.5.3. Depression

This variable was measured through the Yesavage Geriatric Depression Scale, a hetero-administered questionnaire used to detect depression in people over 65 years of age and which has been validated in Spanish [28]. In this study, the abbreviated version of 15 questions has been used, which are answered affirmatively or negatively, taking into account how the subject has felt in the previous week [29]. The scale focuses on the cognitive and behavioral aspects linked to the particularities of the elderly related to depression. It can be completed in 5 to 7 min and the maximum score is 15 points, of which 10 indicates depression when answered affirmatively and 5 indicates depression when answered negatively. A score between 0–4 is considered normal, 5–8 shows mild depression, 9–11 shows moderate depression and 12–15 shows severe depression.

#### 2.5.4. Anxiety

Anxiety was measured by the Hamilton Scale [30] used to detect anxiety by exploring the interruption of the emotional continuum and subjective sensations of tension, restlessness, or nervousness. This scale is made up of 14 items that assess physical, mental and behavioral symptoms characteristic of anxiety, evaluating both their frequency and intensity, of which 7 measure psychological anxiety (1–6 and 14) and the other 7 somatic anxiety (7–13), evaluated from 0 to 4 following the scale 0 = Absent, 1 = slight, 2 = moderate, 3 = severe and 4 = very serious. The total score ranges between 0 and 56 points. A higher score indicates a higher intensity of anxiety.

#### 2.5.5. Quality of Life

The SF-12 Generic Health Questionnaire is the reduced version of the SF-36 that measures aspects related to the functional and emotional state of the person [31]. This self-administered questionnaire, with a completion time of 2 min, is made up of a total of 8 dimensions that refer to both the physical and mental state of the participants: physical function, mental health, general health, emotional role, bodily pain, physical role, social function and vitality. In addition, the sum of both the physical domains and the mental domains can be calculated: physical summary (PCS) and mental summary (MCS). The number of responses to each question ranges between 3 and 5, depending on the item, collecting both positive and negative information related to health. Each item ranges from 0 to 100 points, with higher being a better quality of life.

### 2.6. Sample’s Size Calculation

The sample size was calculated with Jan 3.0 (GlaxoSmithKline, SA, Madrid, Spain). The sample size for this study was 34 subjects per group; this sample was calculated considering an expected increase of 1.5 points on the MMSE [32] detectable with a significance level of 5% and a statistical power of 80%, with a standard deviation of ≤2.5 points, a dropout rate of 35% and a correlation coefficient of 0.650.

### 2.7. Statistic Analysis

The statistical analysis was carried out using the SPSS statistical program, version 20.0 for Windows (SPSS, Inc., Chicago, IL, USA). We worked with a level of statistical significance of *p*-value < 0.05. The continuous variables were presented by means and standard deviations and the categorical variables in frequencies and percentages. The Kolmogorov–Smirnov test was used to check the normality of the data distribution. To determine the possible differences between both study groups before the start of the study, a Student’s *t*-test and chi-square test were used for continuous and categorical variables, respectively. To analyze the possible differences in values between the variables studied, a mixed analysis of variance was carried out, in which the study group was considered to be the intergroup factor (CG vs. EG), and the measurement time of the variables (pre- and post-intervention) the intragroup factor. Dependent variables were measurements obtained on the MMSE for cognitive performance, the Yesavage Geriatric Depression Scale, the Hamilton Scale for Anxiety, and the SF-12 Generic Health Questionnaire. All the analyses were carried out independently for each dependent variable and the possible interactions “group x measurement time” were analyzed. To assess the effect size of the possible intergroup and intragroup differences, Cohen’s d statistic was used. Values of <0.2 indicated an insignificant effect size, between ≥0.2 and <0.5 a small effect size, between ≥0.5 and <0.8 a medium effect size, and values ≥ 0.8 a large effect size [33].

## 3. Results

Table 1 shows the baseline characteristics of the participants at the beginning of the study; no significant differences were found between the groups. All the participants completed at least 92.3% of the sessions and no injuries or adverse effects were reported during the course of the intervention.

### 3.1. Cognitive Performance

According to our results, higher (and therefore better) scores were obtained in the experimental group after the cognitive stimulation program (t(35) = −3.389, *p* = 0.002, Cohen’s d = 0.06). In addition, differences were observed between the groups after the intervention (t(69) = −2.120, *p* = 0.38, Cohen’s d = 0.07) (Table 2).

### 3.2. Cognitive Impairment

According to our results, higher (and therefore better) scores were obtained in the group that underwent the cognitive stimulation program (t(35) = −3.749, *p* = 0.001, Cohen’s d = 0.58). In addition, differences were observed between the groups after the intervention (t(69) = −2.448 *p* = 0.017, Cohen’s d = 0.48) (Table 2).

### 3.3. Depression

According to our results, lower (and therefore better) scores were obtained in the experimental group after the cognitive stimulation program (t(35) = 3.140, *p* = 0.003, Cohen’s d = 0.50). In addition, differences were observed between the groups after the intervention (t(69) = 2.113, *p* = 0.038, Cohen’s d = 0.24) (Table 2).

### 3.4. Anxiety

According to our results, lower (and therefore better) scores were obtained in the group that carried out the cognitive stimulation program (t(35) = 4.921, *p* = 0.000, Cohen’s d = 0.25). In addition, differences were observed between the groups after the intervention (t(69) = 2.134, *p* = 0.036, Cohen’s d = 0.39) (Table 2).

### 3.5. Quality of Life

Table 3 shows the quality of life values before and after the intervention. A detailed analysis of group × time interactions revealed significant differences between measurement times for the cognitive stimulation group and between both groups after the intervention in the domains general health (t(35) = −6.598, *p* = 0.000, d = 0.72 and t(69) = −2.999, *p* = 0.000, d = 0.98); emotional role (t(35) = −2. 256, *p* = 0.030, d = 0.59 and t(69) = −2.915, *p* = 0.006, d = 0.70); social functioning (t(35) = −4.125, *p* = 0.000, d = 0.50 and t(69) = −3.327, *p* = 0.001, d = 0.79); vitality (t(35) = −7.249, *p* = 0. 000, d = 0.81 and (t(69) = −2.086, *p* = 0.041, d = 0. 50); mental health (t(35) = −4.498, *p* = 0.000, d = 0.90 and t(69) = −5.500, *p* = 0.000, d = 1.31) and MCS (t(35) = −8.338, *p* = 0.000, d = 1.26 and t(69) = −5.126, *p* = 0.000, d = 1.22).

However, there were no significant results with respect to physical functioning, bodily pain, physical role, or PCS.

## 4. Discussion

The aim of this study was to analyze the effect of a 12-week cognitive stimulation program on cognitive performance, anxiety, depression and HRQoL in people aged > 70 and with mild cognitive impairment. Our results revealed that cognitive performance, anxiety, depression, and psychological status on the SF-12 scale had improved.

The increasing prevalence of cognitive impairment at older ages has encouraged the study of new nonpharmacological treatments for its prevention or maintenance [34]. In the present study, participants who received a cognitive stimulation program for 12 weeks showed an improvement in cognitive performance, but there was no change between the ranges obtained in both the MMSE and the MoCA. In agreement with our results, a meta-analysis concluded that cognitive training improves cognitive performance in older people with mild cognitive impairment [35] and a study found that a computerized cognitive stimulation program influenced cognitive performance [36], but the design of these studies (quasi-experimental) did not allow comparisons with a control group. On the other hand, Hughes et al. [37] found that training with interactive video games improved cognitive and physical function, but the difference was not statistically significant.

Depression is a common condition in people with mild cognitive impairment [38] and a recent meta-analysis showed an increased risk of developing cognitive pathologies in people with a history of depression [39]. Furthermore, depression, anxiety, and other negative mental health symptoms have been shown to significantly reduce cognitive functioning in older people with MCI [40]. The implementation of interventions to slow the progression of cognitive impairment and depressive states in the elderly is particularly important. Our findings showed that people who received cognitive stimulation training had improvements in depression. This finding may be due to new friendships and exposure to others with similar problems. Similar results have been reported previously after cognitive training, but in longer intervention programs (16 weeks) [41] and in patients with mild cognitive impairment in health centers and nursing homes [42].

There is evidence supporting significant associations between anxiety and mild cognitive impairment [43] and a systematic review suggested that psychological treatments have positive effects in reducing anxiety-related symptoms in people with MCI [44]. Regarding the cognitive stimulation program, decreases in anxiety symptoms have been reported in healthy elderly populations [45]. Our findings showed that people with MCI who were enrolled in a cognitive stimulation program experienced improvements in anxiety after the intervention period compared with a control group. Similarly, the study by Talassi et al. [46] observed statistically significant differences in the short-term evaluations, but unlike our study, with another type of intervention such as a computerized cognitive rehabilitation program and using the State-Trait Anxiety Inventory (STAI) for the level of anxiety. However, other works that have carried out an intervention similar to ours, such as Gomez-Soria et al. [32], found no statistically significant differences in anxiety levels measured by the Goldberg scale in older people with mild cognitive impairment. Furthermore, this study found no significant improvements in depression, another variable studied in our study, and its intervention lasted only 10 sessions compared to our study, which carried out a total of 36 sessions. Therefore, our study can be considered as a novel contribution in this type of population.

Finally, HRQoL is a fundamental issue in the context of attention to cognitive impairment, based on studies such as Muangpaisan et al. [47], mild cognitive impairment has a negative association with HRQoL, specifically mental quality of life. In this study, participants who completed the cognitive training program showed benefits in the SF-12 mental summary, but no improvement in the physical summary. Similarly, there are studies that report improvements in HRQoL, but with another type of cognitive training, i.e., a study in elderly people with cognitive impairment showed improvements in HRQoL as assessed by the QOL questionnaire after computer-based cognitive training [48]. On the other hand, the efficacy of different types of physical activity on the quality of life of people with MCI has also been demonstrated and shows an improvement in both, physical and mental summary [49]. Therefore, new studies that evaluate the benefits of a combined physical–cognitive program for the improvement of this variable should be considered for comparison with previous studies.

Despite the results of our study, some of its limitations should be acknowledged: only short-term effects were evaluated. Although participants were blinded to the hypothesis under test, they were well aware of the intervention itself and, therefore, a self-report bias is a distinct possibility. Finally, information on possible related biomarkers has not been included in the study. Future studies should consider long-term effects and take into account possible associated biomarkers.

## 5. Conclusions

In conclusion, this study demonstrated that a 12-week cognitive stimulation program has a beneficial effect on cognitive performance, anxiety, and depression in people with mild cognitive impairment and over 70 years of age. Improvements were also seen in the mental domains of quality of life, specifically emotional role, social function, vitality, and mental health, as well as in general health. These results have important clinical implications for the target population, since it opens new possibilities to delay cognitive deterioration, thus improving their mental health and quality of life.

## Figures and Tables

**Table 1:** Baseline characteristics of study participants.

		Total (*n* = 71)	EG (*n* = 36)	CG (*n* = 35)	*p*-Value
Age (years)		75.08 ± 3.82	75.36 ± 3.74	74.80 ± 3.92	0.540
Sex	Male	23 (32.39)	25 (52.10)	23 (47.90)	0.741
	Female	48 (67.61)	11 (47.80)	12 (52.20)	
Marital status (%)	Single	10 (14.08)	5 (50.00)	5 (50.00)	0.983
	Married	43 (60.56)	22 (51.20)	21 (48.80)	
	Separated/widowed	18 (25.35)	9 (50.00)	9 (50.00)	
Education	No formal education	15 (21.13)	8 (53.3)	7 (46.7)	0.500
	Primary education	41 (57.75)	22 (53.7)	19 (46.3)	
	Secondary education	6 (8.45)	2 (33.3)	4 (66.7)	
	University	9 (12.68)	4 (44.4)	5 (55.6)	
MMSE		25.11 ± 0.82	25.08 ± 0.84	25.14 ± 0.81	0.762
MoCA		20.45 ± 2.26	20.39 ± 2.42	20.51 ± 2.12	0.817
Depression		6.24 ± 3.30	6.00 ± 3.14	6.49 ± 3.48	0.539
Anxiety		19.17 ± 11.07	19.83 ± 11.94	18.49 ± 10.24	0.612
SF-12	General health	58.94 ± 20.19	56.53 ± 21.97	61.43 ± 18.17	0.310
	Physical functioning	74.37 ± 23.33	75.00 ± 23.42	73.71 ± 23.56	0.818
	Physical role	71.83 ± 38.48	66.67 ± 41.40	77.14 ± 35.03	0.254
	Emotional role	94.84 ± 16.60	92.59 ± 17.70	97.14 ± 12.45	0.250
	Social functioning	87.94 ± 19.95	87.28 ± 20.69	88.63 ± 19.44	0.778
	Bodily pain	69.78 ± 21.86	71.56 ± 20.84	67.96 ± 23.02	0.492
	Vitality	62.32 ± 20.89	57.64 ± 18.92	67.14 ± 21.97	0.055
	Mental health	68.03 ± 20.21	68.44 ± 23.44	67.60 ± 21.20	0.874
	PCS	68.73 ± 13.25	67.44 ± 13.62	70.06 ± 13.62	0.408
	MCS	78.28 ± 10.32	76.49 ± 10.40	80.13 ± 10.05	0.138

Data are expressed as mean and standard deviation and frequency and percentage for continuous or categorical variables, respectively. EG: experimental group; CG: control group; MMSE: Mini-Mental State Examination; MoCA: The Montreal Cognitive Assessment; SF-12: 12-Item Short-Form Health Survey. PCS: Physical component summary. MCS: mental component summary.

**Table 2:** Effects of the cognitive stimulation program on cognitive performance, cognitive impairment, anxiety and depression.

	EG (*n* = 26)		CG (*n* = 24)		Group			Time			Group × Time		
	Pre	Post	Pre	Post	F(1.69)	*p*-Value	η^2^	F(1.69)	*p*-Value	η^2^	F(1.69)	*p*-Value	η^2^
MMSE	25.08 ± 0.84	25.44 ± 0.74	25.14 ± 0.81	25.09 ± 0.80	0.698	0.406	0.010	5.603	0.021	0.075	10.609	0.002	0.133
MoCA	20.39 ± 2.42	21.53 ± 2.26	20.51 ± 2.12	20.23 ± 2.21	1.400	0.241	0.020	4.469	0.038	0.061	12.460	0.001	0.153
Depression	6.00 ± 3.14	5.31 ± 2.69	6.49 ± 3.48	6.80 ± 3.23	1.865	0.177	0.026	1.126	0.292	0.016	7.927	0.006	0.103
Anxiety	19.83 ± 11.94	15.75 ± 8.60	18.49 ± 10.24	19.49 ± 18.68	0.246	0.621	0.004	10.614	0.002	0.133	28.850	0.000	0.295

The quantitative variables are presented as mean and standard deviation. Qualitative variables are presented as frequency and percentage. EG: experimental group; CG: control group; MMSE: Mini-Mental State Examination; MoCA: The Montreal Cognitive Assessment.

**Table 3:** Effects of the cognitive stimulation program on quality of life.

	EG (*n* = 26)		CG (*n* = 24)		Group			Time			Group × Time		
	Pre	Post	Pre	Post	F(1.69)	*p*-Value	η^2^	F(1.69)	*p*-Value	η^2^	F(1.69)	*p*-Value	η^2^
General health	56.53 ± 21.97	70.97 ± 18.16	61.43 ± 18.17	52.86 ± 18.68	0.898	0.347	0.013	13.080	0.001	0.159	35.911	0.000	0.342
Physical functioning	75.00 ± 23.42	74.86 ± 22.09	73.71 ± 23.56	74.00 ± 21.52	0.041	0.840	0.001	0.006	0.941	0.000	0.046	0.830	0.001
Physical role	66.67 ± 41.40	62.50 ± 45.32	77.14 ± 35.03	64.29 ± 44.67	0.424	0.517	0.006	7.304	0.009	0.096	1.903	0.172	0.027
Emotional role	92.59 ± 17.70	100.00 ± 0.00	97.14 ± 12.45	93.33 ± 13.53	0.195	0.660	0.003	0.722	0.399	0.010	7.015	0.10	0.92
Social functioning	87.28 ± 20.69	95.88 ± 12.57	88.63 ± 19.44	85.04 ± 14.75	1.662	0.202	0.024	2.013	0.160	0.028	11.899	0.001	0.147
Bodily pain	71.56 ± 20.84	83.14 ± 19.30	67.96 ± 23.02	73.27 ± 86.18	0.672	0.415	0.010	1.309	0.256	0.019	0.180	0.673	0.003
Vitality	57.64 ± 18.92	71.94 ± 16.14	67.14 ± 21.97	62.57 ± 21.30	0.000	0.988	0.000	9.451	0.003	0.120	35.544	0.000	0.340
Mental health	68.44 ± 23.44	85.89 ± 13.94	67.60 ± 21.20	61.83 ± 21.93	9.481	0.003	1.21	4.739	0.33	0.064	17.744	0.000	0.214
PCS	67.44 ± 13.62	72.87 ± 13.25	70.06 ± 13.62	66.03 ± 26.39	0.353	0.555	0.005	0.108	0.744	0.002	4.905	0.030	0.066
MCS	76.49 ± 10.40	87.35 ± 6.29	80.13 ± 10.05	78.20 ± 8.55	2.063	0.155	0.029	23.560	0.000	0.255	48.394	0.000	0.412

The quantitative variables are presented as mean and standard deviation. Qualitative variables are presented as frequency and percentage. EG: experimental group; CG: control group. PCS: physical component summary. MCS: mental component summary.

## Data Availability

The data shown in this study are available upon request from the corresponding author. The data are not available to the public, since taking into account the sensitive nature of all the questions asked in this study, all participants were guaranteed that the data obtained would be confidential and would not be shared.
